# Superparamagnetic iron oxide nanoparticles for their application in the human body: Influence of the surface

**DOI:** 10.1016/j.heliyon.2023.e16487

**Published:** 2023-05-25

**Authors:** Chiara Turrina, Anna Klassen, Davide Milani, Diana M. Rojas-González, Gerhard Ledinski, Doris Auer, Barbara Sartori, Gerhard Cvirn, Petra Mela, Sonja Berensmeier, Sebastian P. Schwaminger

**Affiliations:** a Chair of Bioseparation Engineering, TUM School of Engineering and Design, Technical University of Munich, Germany; b Chair of Medical Materials and Implants, TUM School of Engineering and Design, Munich Institute of Biomedical Engineering, Technical University of Munich, Germany; c Division of Medicinal Chemistry, Otto Loewi Research Center, Medical University of Graz, Austria; d Division of Medical Physics and Biophysics, Gottfried Schatz Research Center, Medical University of Graz, Austria; e Institute of Inorganic Chemistry, Graz University of Technology, Stremayrgasse 9/IV, Graz, 8010, Austria; f BioTechMed-Graz, Austria

**Keywords:** Iron oxide nanoparticles, Nanomedicine, Simulated body fluids, Cytocompatibility, Agglomeration, Magnetic separation

## Abstract

Iron oxide nanoparticles (IONs) are of great interest in nanomedicine for imaging, drug delivery, or for hyperthermia treatment. Although many research groups have focused on the synthesis and application of IONs in nanomedicine, little is known about the influence of the surface properties on the particles' behavior in the human body. This study analyzes the impact of surface coatings (dextran, polyvinyl alcohol, polylactide-co-glycolide) on the nanoparticles’ cytocompatibility, agglomeration, degradation, and the resulting oxidative stress induced by the particle degradation. All particles, including bare IONs (BIONs), are highly cytocompatible (>70%) and show no significant toxicity towards smooth muscle cells. Small-angle X-ray scattering profiles visualize the aggregation behavior of nanoparticles and yield primary particle sizes of around 20 nm for the investigated nanoparticles. A combined experimental setup of dynamic light scattering and phenanthroline assay was used to analyze the long-term agglomeration and degradation profile of IONs in simulated body fluids, allowing fast screening of multiple candidates. All particles degraded in simulated endosomal and lysosomal fluid, confirming the pH-dependent dissolution. The degradation rate decreased with the shrinking size of particles leading to a plateau. The fastest Fe^2+^ release could be measured for the polyvinyl-coated IONs. The analytical setup is ideal for a quick preclinical study of IONs, giving often neglected yet crucial information about the behavior and toxicity of nanoparticles in the human body. Moreover, this study allows for the development and evaluation of novel ferroptosis-inducing agents.

## Highlights

• Effects of various coatings on the behavior of iron oxide nanoparticles.
• Agglomeration and saturation magnetization influence their magnetophoretic behavior.
• Fast experimental setup for screening the nanoparticles for usage in nanomedicine.
• Usage of simulated body fluids.
• Agglomeration and degradation directly affect each other.

## Graphical abstract

**Figure:**
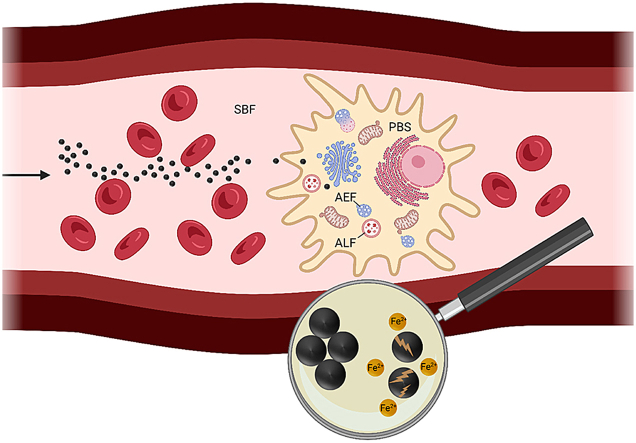

## 1 Introduction

One of nanotechnology's most active research fields is nanomedicine, which applies nanotechnology to precise medical interventions for preventing, diagnosing, and treating diseases [1,2]. With 76% of scientific papers and 59% of patents, drug delivery is the most dominant part of nanomedicine [3]. Iron oxide nanoparticles (IONs) can be applied for targeted or magnetically controlled drug delivery [4]. Additional to their superparamagnetic behavior, high surface-to-volume ratio, fast and cost-efficient synthesis, and low toxicity, IONs can encapsulate, disperse, adsorb, or conjugate drugs [5]. Guided by an external magnetic field, drug-loaded particles can be specifically transported to unhealthy tissue, like cancerous tumors, thereby reducing systemic side effects and decreasing the overall amount of drugs [6,7]. To date, intravenous injection of drug-loaded nanoparticles is one of the most widely explored systems in targeted drug delivery [8]. IONs are already well accepted as T2 (transverse relaxation time) contrast agents for noninvasive magnetic resonance imaging (MRI). The superparamagnetic core of IONs affects the transverse relaxation time of protons in nearby tissues and can be measured by a darkened tissue [9]. IONs have also found an application in hyperthermia, an alternative cancer treatment [10]. Here, an alternating magnetic field is used to heat the temperature-sensitive tumor cells to temperatures ≥41 °C [10].

For the application in nanomedicine, IONs must be biocompatible, colloidally stable, and often need to be specifically functionalized [[10], [11], [12]]. These criteria are usually not fulfilled by bare IONs (BIONs), leading to massive research in nanoparticle coatings in recent years [11,12]. Often a core-shell structure is preferred, leading to stabilized and flexible particle systems [10]. Organic coatings are advantageous due to their biocompatibility and water solubility [12,13]. Polyethylene glycol (PEG), polyvinylpyrrolidone (PVP), polyvinyl alcohol (PVA), chitosan, and dextran (Dex) are well known to prolong the circulation of particles in the blood [[12], [13], [14], [15]]. For this study, poly(lactid-co-glycolid) (PLGA), Dex, and PVA-coated IONs are analyzed as they are already established and commonly used coatings [11].

Dex-coated IONs are used as contrast agents in MRI scans and to treat anemia in patients [16,17]. For drug delivery application, Khalkhali et al. were able to bind and slowly release a polyphenolic plant constituent (curcumin) with pharmacological properties from Dex-coated IONs [18]. Unterweger et al. and Peng et al. also investigated the use of ION@Dex as a drug delivery system in cancer therapy by loading the particles with the chemotherapeutic drug doxorubicin or hypericin [19,20]. For some of the described applications, the Dex-coating has already been approved by the Food and Drug Administration (FDA) [16,17,21]. PLGA is biodegradable. It can be hydrolyzed to the metabolic monomers lactic acid and glycolic acid [22]. Furthermore, PLGA as material is approved by the FDA and European Medicines Agency (EMA) [22,23]. ION@PLGA can encapsulate hydrophobic and hydrophilic molecules and protect the drug from degradation, making it ideal for drug delivery systems [22]. Saengruengrit et al. used ION@PLGA to deliver proteins to bone marrow-derived primary dendritic cells [24]. Ruggiero et al. synthesized ION@PLGA particles loaded with multiple anticancer drugs that perform a magneto fluid hyperthermia-triggered drug release [25]. PVA has a hydrophilic character and is biodegradable [26]. It is widely used as a protective agent to stabilize IONs and is approved by the FDA [11,27]. Kayal et al. confirmed that ION@PVA is a promising candidate for magnetically targeted drug delivery of doxorubicin [28]. Furthermore, Ebadi et al. used PVA/LDH (magnesium-aluminium-layered double hydroxide)-coated particles with the drug sorafenib and showed high drug release in simulated acidic tumor environments [29].

For an application in the human body, degradation and colloidal stability over time are critical factors to consider in the preclinical development of IONs [30]. Thus long-term agglomeration and Fe^2+^-release studies are essential to better understand the particles' behavior after injection into the body. The latter is important since the degradation of IONs, depending on the coating, can be very slow, with a half-life of several weeks [31]. The morphology, size, and agglomeration behavior influence the degradation of particles [32]. Smaller particles dissolve faster than large ones [32]. Long-term studies in mice showed that maghemite nanoparticles were degraded in the lysosome and were ultimately stored in the liver and the spleen [31]. Levy et al. demonstrated that colloidal stability due to the degradation profile of three differently coated IONs is influenced by the pH and they identified a degradation optimum for these particles at pH 4 [33]. Undesirable side effects can occur if the particles agglomerate in the body. For biomedical applications, particles smaller than 100 nm are recommended to prevent toxic effects such as thrombogenesis and prolonged blood circulation [11]. In addition, Limbach et al. reported that slight variations in the particle's surface chemistry significantly impact the stability, slow down the aggregation rate, and enhance the colloidal stability [34]. Following intravenous injection, the particles encounter a physiological pH of 7.4 in the blood and cytosol to more acidic pH (4–5) in degradation compartments [[35], [36], [37]].

Simulated fluids represent an interesting and cost-efficient alternative to *in vivo* experiments, which have been increasingly used in recent years [36,38]. They allow the conception of fast and effective screenings of potentially applicable and safe nanoparticles in nanomedicine in the preclinical phase. Additionally, animal experiments can be reduced to a minimum [36]. SBF (simulated body fluid, pH = 7.4) simulates the conditions in the blood circulation, and PBS (phosphate buffered saline, pH = 7.4) simulates the cytosol [36,39,40]. Late endosomes and lysosomes degrade the particles within the cells. AEF (artificial endosomal fluid, pH = 5.5) simulates the conditions in the late endosome, and ALF (artificial lysosomal fluid, pH = 4.5) the conditions after particle uptake in the lysosome [41]. The buffers simulate the path a particle can take in the body after intravenous administration. They have a much more complex composition than simple acids and are similar to the original hard-to-reach fluids due to their salt composition [31,35,42,43].

Under these acidic conditions, IONs are easily dissolved. This is especially true for complexing and reducing buffers containing carboxylic acids such as citrate [44]. This results in the formation of iron ions. Iron ions have gained great interest for usage in ferroptosis applications. Especially the combination of ferric and ferrous ions is interesting for use in ferroptosis applications and the generation of reactive oxygen species (ROS) in cells [45]. Thus, multiple nanoparticle-based systems have been developed in the last years to deliver iron ions for ferroptosis therapy [46]. Aside from crystalline materials, amorphous iron nanoparticles and metal organic frameworks (MOFs) have also been developed for the specific release of ferrous and ferric ions [47]. Acidic environments allow for Fenton reactions and hydrogen peroxide production, which can be used in tumor treatment [48]. The general pathway for the treatment is via the production of very reactive hydroxyl radicals, which oxidize lipoproteins. Following this oxidation, lipid peroxides induce ferroptosis [49].

Although many research groups have focused on the synthesis and application of IONs in nanomedicine, little is known about the influence of the surface properties on the particle's behavior in the human body. To better understand the impact of different coating materials on the IONs degradation and agglomeration profile, BIONs, ION@Dex, ION@PVA, and ION@PLGA were incubated in simulated body fluid (SBF), artificial lysosomal fluid (ALF), artificial endosomal fluid (AEF), and phosphate-buffered saline (PBS) for 72 h at 37 °C. The hydrodynamic diameters were analyzed by dynamic light scattering (DLS) and the Fe^2+^ release by phenanthroline assay. Oxidative stress was analyzed with an low-density lipoprotein (LDL) assay. The novelty of this investigation is the release of iron ions in artificial bodily fluids depending on the surface modifications of iron oxide nanoparticles. The presented data gives essential insights into the applicability of IONs in nanomedicine and shows a fast screening method that can be used in the preclinical phase.

## 2 Material and methods

### 2.1 Synthesis

#### 2.1.1 BIONs

BIONs were synthesized by the co-precipitation method according to the Massart process [50]. The experiment was performed analogously to Turrina et al. [51]. The characterization data was previously published and is only used for comparison [52].

#### 2.1.2 Dextran-coated IONs

Dextran-coated particles were prepared similarly to BIONs using the Massart process. In this process, 9.5 mL of Dextran solution (10.0 g L^−1^, Sigma Aldrich) was added with 83 mL of iron (II/III) solution (FeCl_2_ × 4H_2_O (735 mg), Sigma Aldrich; FeCl_3_ × 6H_2_O (2000 mg), Carl Roth) in a 100 mL round bottom flask under N_2_ atmosphere. Co-precipitation is started after adding 7.5 mL of 25% NH_4_OH solution, and the reaction was run under homogeneous stirring at 70 °C for 30 min. After completion of the reaction, the excess salts are removed by washing with degassed ddH_2_O (4×) until a conductivity lower than 200 μS cm^−1^ is obtained.

#### 2.1.3 PVA-coated IONs

The principles of the Massart process were applied for the synthesis of the ION@PVA particles. Beforehand, 2.88 g NaOH (Carl Roth) and 3.00 g PVA (Sigma Aldrich) were mixed in 20 mL degassed water and treated in an ultrasonic bath until everything had dissolved. In parallel, 40 mL of iron (II/III) solution (FeCl_2_ × 4H_2_O (1.40 g), Sigma Aldrich; FeCl_3_ × 6H_2_O (3.47 g), Carl Roth) was prepared in degassed water. The reaction was started by mixing the two solutions in a 100 mL round bottom flask under nitrogen conditions. The reaction was run for 30 min at 80 °C. The reaction broth was washed with absolute ethanol (2×, VWR chemicals) and degassed ddH_2_O (3×) until a conductivity below 200 μS cm^−1^ was achieved.

#### 2.1.4 PLGA-coated IONs

ION@PLGA was synthesized by a single emulsion method [53]. In this preparation technique, 40.0 mg, PLGA 50:50 of 38–54 kDa (Fluka Sigma Aldrich) were dissolved in an organic phase comprised of dichloromethane and acetone at a 2:1 vol ratio. Additionally, 100 μL of ultrasonicated 3-aminopropyltrimethoxysilane (APTS) coated IONs suspended in ethanol (10.0 g L^−1^) were added. Afterward, 6.00 mL of an aqueous phase containing 0.30% PVA (Fluka Sigma Aldrich) was poured into the solution and emulsified by ultrasonication in the Branson Digital Sonifier 450 (Emerson Electric Co, 30 s, 30.0%, 15 s ON, 15 s OFF). Complete evaporation of the organic phase was ensured by continuous stirring at 550 rpm and at a temperature of 25 °C for 17 h. The particles were magnetically separated and washed with 2.00 mL of deionized water (3×) to remove excess PVA and other loosely adsorbed excipients. All particles are stored in deionized water under a N_2_ atmosphere at 4 °C.

### 2.2 Characterization

(Fourier-transform infrared spectroscopy) FT-IR spectroscopy (Alpha II; Bruker Corporation; Billerica) and platinum attenuated total reflection module (4000 cm^−1^ to 400 cm^−1^, 24 scans) were used to confirm functional groups' presence on the IONs’ surface. The background was subtracted with the concave rubber band method (OPUS 8.1). Lyophilized IONs (Alpha 1–2 Ldplus, Christ, −60 °C overnight in vacuum) were analyzed by powder X-ray diffraction (XRD) with the STOE Stadi-P Diffractometer (flatbed measurement, molybdenum source, 0.7093 Å). The saturation magnetization was determined by SQUID analysis. 10 mg of particles were fixed in a small plastic tube (Fixogum, Marabu GmbH & Co KG, Tamm, Germany) and measured with the magnetometer MPMS XL-7 (Quantum Design, San Diego USA) at 300 K and a magnetic field variation of −50 kOe to +50 kOe. The LUMIReader (4532-123; LUM GmbH) was used to analyze the sedimentation rate of the IONs in a magnetic field by STEP technology. The particles (1.00 g L^−1^, pH 7) were ultrasonicated and then contacted with five stacked cylindrical neodymium (NdFeB) magnets (d = 12 mm; h = 2 mm, N45, Webcraft GmbH, Gottmadingen, Germany) and measured at wavelengths of 870 nm, 630 nm, and 420 nm (Profile: 1000; Interval: 1s; Angle: 0°; Light factor: 1.00; Temperature: 25 °C; magnetization 29.1–54.4 Am^2^ kg^−1^). The processing of the obtained data was performed by the software PSA-Wizard (SEPview™; Analysis positions: 13.0 mm, 15.0 mm, 17.0 mm, 19.0 mm). The particle size and morphology are measured by Transmission electron microscopy (TEM) with the TEM JEM JEOL 1400 (×120k). The samples (0.03 g L^−1^, 10 μL, after ultrasonication) were dried onto a glow-discharged carbon-coated copper grid. For analysis (ImageJ), at least 100 particles from three different areas were measured. The Zetasizer Ultra (Malvern Panalytical) was used to measure dynamic light scattering (DLS) and the zeta potential of a 1 g L^−1^ solution (ddH_2_O pH = 2–10, after ultrasonication, Cuvetta STD UV 4 clear side, KARTELL S. p.a., and DTS1070, Malvern Instruments). A Boltzmann fit determined the isoelectric point (IEP).

Small angle X-ray scattering (SAXS) data were acquired at the Austrian SAXS beamline at the Elettra Synchrotron in Trieste; the beamline length was set to 1386.101 mm, corresponding to a q range of 0.07 nm^−1^ - 5.3 nm^−1^, where q = 4π sinθ/λ, λ is the wavelength of the incident X-rays, and 2θ is the scattering angle. The photon energy was set to 8 keV corresponding to a wavelength of 0.154 nm. The sample was loaded in a quartz capillary with 1.5 mm diameter and exposed to X-rays. 10 images of 10 s each were collected by a Pilatus 3 1 M detector (Dectris Ltd., Baden, Switzerland). The angular scale of the diffraction pattern was calibrated with silver behenate (d-spacing 5.8376 nm). The acquired images were azimuthally integrated by SAXSDog, the automatic data integration pipeline available at the SAXS outstation, normalized on transmission and fluctuation of the primary beam intensity, and background subtracted.

The experimental setup for optofluidic force induction (OF2i) measurements consists of a 2D optical trap in a cylindrical flow cell using a weakly focused doughnut-shaped vortex beam. The laser beam is generated by a 532 nm linear polarized CW DPSS laser (Laser Quantum, GEM532) with a maximum output power of 2 W. The beam alignment is achieved using two mirrors and a 5× expander. A vortex phase plate generates a Laguerre-Gaussian mode with topological charge m = 2. The ultramicroscope consists of a 10× PLAN microscope objective, an optical filtering bank, a 50 mm focusing lens, and a CCD camera for imaging. The microfluidic flow cell consists of a continuous, dead volume optimized pumping and laminar fluid handling setup, following derivable fluid continuity principles [54]. Particles have been dispersed in acetate buffer solutions and ultrasonicated at concentrations of around 1 μg mL^−1^ prior to OF2i measurements.

Human low-density lipoprotein (LDL) was obtained from the plasma of normolipemic, fasting male donors by sequential ultracentrifugation within the density of 1.020–1.050 g mL^−1^. Diethylenetriaminepentaacetic acid (DTPA) and Pefabloc were present during all steps of lipoprotein preparation to prevent lipid peroxidation and apo B cleavage by contaminating bacteria or proteinases. The LDL were dialysed against a 10 mM Tris HCl, Isotone, 100 μM DTPA, pH 7.40, sterile-filtered and stored at 4 °C in the dark until use. The LDL concentration was measured by dry weight determination and the protein content by the Lowry method [55]. The freshly prepared LDL were dialysed against a 10 mM PBS without DTPA. Subsequently, the LDL was diluted with a 100 mM sodium acetate buffer, pH 4.50 to give a concentration of 0.2 mg LDL mL^−1^. The nanoparticle suspensions (1 g L^−1^) were added to the LDL to give a final concentration of 200 μM. The formation of conjugated dienes was continuously monitored at 234 nm by a spectrophotometer (Hitachi U-2000) at 37 °C for 240 min using 1 cm quartz cuvette [56].

Cytocompatibility was assessed following ISO-10993 guidelines. Human umbilical artery smooth muscle cells (HUASMCs, Promocell, passage 7) were used for the cytocompatibility assay. The cells were expanded in culture medium consisting of DMEM supplemented with 10% fetal calf serum (FCS, Gibco) and 1% antibiotic/antimycotic mix (ABM, Gibco). The different IONs were sterilized using H_2_O_2_-low-temperature-plasma, resuspended in the phosphate-buffered saline, ultrasonicated (10 min), and transferred to the medium by magnetic separation. The samples were diluted to 0.1 g L^−1^ in culture medium. For the test, 10.000 cells cm^−2^ were seeded in 96-well plates (Greiner Bio-One, Germany) and incubated at 37 °C with 5% CO_2_ for 24 h to allow for cell adhesion. Afterward, the different experimental samples were added to the cells. Culture medium served as the negative control, while culture medium supplemented with 2% Triton x-100 (Sigma) was used as the positive control. Cell proliferation was analyzed 24 and 72 h after adding IONs, by a commercial cell proliferation kit (XTT, Roche) following manufacturer's instructions. Briefly, 50 μL of the working solution (1:50 electron coupling reagent (ECR) with the XTT solution) were transferred to each well and incubated for 2 h. The optical density of formazan was measured at a wavelength of 450 nm and a reference wavelength of 630 nm using a plate reader (Spark, Tecan). An initial measurement at 0 h was used to exclude the effect of the IONs on the optical density. For better data visualization, all values were normalized to the absorbance of the NC.

The composition of PBS, AEF, ALF, and SBF can be found in the supplementary material (Tables S1–S3).

A 1.00 g L^−1^ particle solution in water (BIONs, ION@PVA, ION@Dex, ION@PLGA) was prepared and ultrasonicated (30 min). For each buffer, triplicates were prepared by exchanging the liquid phase through magnetic decantation. All samples are incubated at 37 °C in an incubation shaker (Thermomixer comfort, Eppendorf) at 1000 rpm for 72 h. The hydrodynamic diameters were measured by DLS (Cuvetta STD UV 4 clear side, KARTELL S.p.a.) using Zetasizer Ultra (Malvern Panalytical) at 37 °C after 0, 1, 3, 5, 24, 48, and 72 h.

For each buffer, 2.00 mL of a 1.00 g L^−1^ for all particles were prepared as described above and incubated at 37 °C in a thermoshaker at 1000 rpm. After 0, 1, 3, 5, 24, 48, and 72 h, 80 μL of the particle solution were taken and centrifuged at 17,000 rpm for 20 min to remove interfering particles. 60 μL of the supernatant were transferred to a new reaction tube, and 100 μL of 10% (w/v) ascorbic acid (Table S4) and 400 μL of acetate buffer (Table S5) were added. Fe^3+^ ions are reduced to Fe^2+^ by the added ascorbic acid. After incubation for 5 min at room temperature, 50 μL of 0.5% (v/v) phenanthroline solution (Table S6) were added and incubated for another 20 min. Subsequently, the volume is filled up to 1.00 mL with deionized water. Fe^2+^ ions form a red-orange chelate complex with 1,10-phenanthroline [57]. 300 μL of the sample were pipetted into a 96-well plate; then, the absorbance was measured at 510 nm using Tecan Infinite M200 microplate reader. The analysis was performed in triplicates. For the calibration curve, a 0.10 g L^−1^ Fe^2+^ stock solution was prepared from FeCl_2_ × 4H_2_O (Table S7). From the solution 0, 1, 2, 4, 6, 10, 20, 30, 50, 60, 80, 100, 120 and 160 μL were taken. The concentration of free iron ions was normalized to the initial concentration of the particulate stock solution. Herefore, 60 μL of the 300 μL remaining particle solutions were dissolved in 60 μL of concentrated hydrochloric acid (37%). The solution was filled to 1.00 mL with ddH_2_O and mixed. From the diluted solution, 60 μL were treated in the acetate buffer-ascorbic acid mix analogous to the supernatants described above and then mixed with phenanthroline.

## 3 Results and discussion

The co-precipitation technique was used to synthesize BIONs, ION@PVA, and ION@Dex [50,51]. The PLGA coating was generated by the single emulsion method [53].

All particles were analyzed regarding their particle composition, size, surface properties, agglomeration, saturation magnetization, magnetophoretic behavior, and cytocompatibility. The detailed characterization of the used BIONs can be found in Turrina et al. [52].

The successful Dex, PVA, or PLGA coating is determined by FT-IR (Fig. S1). All particles show the characteristic Fe–O peak at 582 cm^−1^ [51,[58], [59], [60]]. For ION@Dex, the spectra showed νC-H at 1622 cm^−1^ and νC-O vibrations at around 1019 cm^−1^ and 1028 cm^−1^ [18,61]. Between 765 cm^−1^ and 914 cm^−1,^ vibrations of the glucopyranose ring can be observed [62]. For PVA-coated IONs, a C–C stretching vibration at 1416 cm^−1^ and a Fe–O–C bond at 1092 cm^−1^ were observed [28]. At 850 cm^−1^, the ρCH_2_ vibration is visible, and adsorbed water was also detected at 1620 cm^−1^ [28]. For ION@Dex and ION@PVA, the intensity ratio of coating and ION is comparable, whereas ION@PLGA shows intense characteristic polymer peaks that overlay the distinct iron oxide peak. This ratio indicates a thicker polymer layer for ION@PLGA than the other two. The peak at 1754 cm^−1^ is attributed to the vibration of the carbonyl groups in the two monomers of PLGA. The bands between 1271 cm^−1^ to 1087 cm^−1^ are assigned to C–O vibrations [63].

**Fig. 1:**
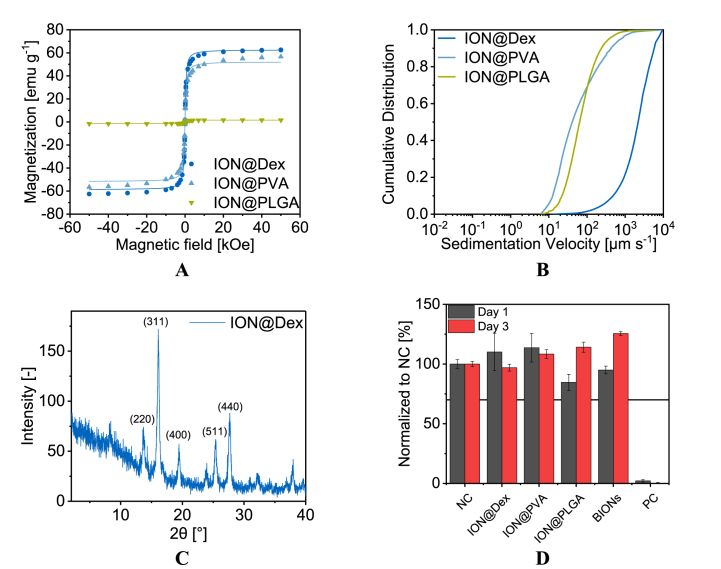
SQUID analysis at 300 K, processed with the Langevin Mod fit (A). Cumulative velocity distribution at pH 7 in water, at room temperature (B). X-ray diffractogram of ION@Dex (C). Cytocompatibility over three days was analyzed by XTT assay with smooth muscle cells on the different particles, as well as a negative control (NC) and a positive control (PC). The results were normalized to NC (D).

The crystal structure of the different particles is determined by X-ray diffraction (XRD) analysis (Fig. 1D, Fig. S2) The measurements show the characteristic reflections corresponding to spinel structured iron oxide at 13.7° (220), 16.1° (311), 19.5° (400), 25.4° (511), and 27.7° (440) for all particles [11]. The coatings do not influence the crystal structure of the IONs.

The particle size is an essential criterion in nanomedicine [64]. A diameter between 10 and 100 nm is ideal for most applications in this field, avoiding rapid cleaning from the bloodstream and easy uptake through the blood-brain barrier [64,65]. Furthermore, small diameters lead to a large surface-to-volume ratio, giving the possibility of binding vast amounts of drugs or presenting many functional groups [11,12].

**Fig. 2:**
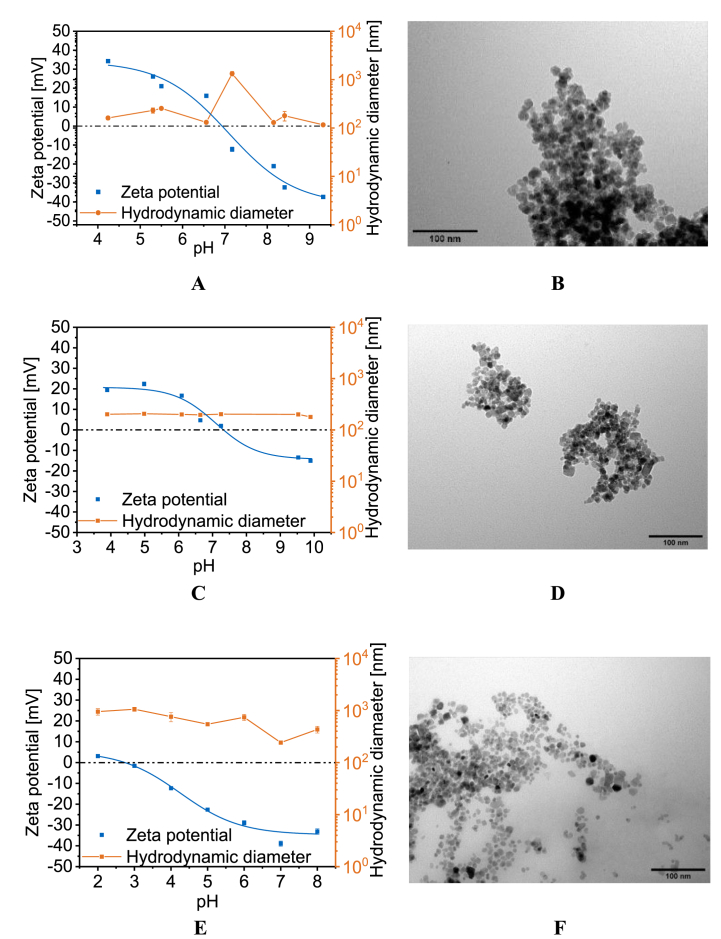
IEP points of ION@Dex (A), ION@PVA (C), and ION@PLGA (E). TEM images of ION@Dex (B), ION@PVA (D), and ION@PLGA (F) at 120kx.

The Scherrer equation (Equation S1) uses XRD data to determine the iron oxide core size. Additionally, the morphology and particle diameter were determined with TEM. The diameters of BIONs coincide, d_Scherrer_ = 8.8 ± 0.9 nm and d_TEM_ = 8.7 ± 1.6 nm [52]. Therefore, the coating thickness was calculated by subtracting d_Scherrer_ from d_TEM._^.^ION@Dex and ION@PLGA have comparable diameters with d_Scherrer=_∼8.7 nm and d_TEM_ = ∼10.5 nm (Table). The calculated coating thickness is, therefore, Δd = ∼1.8 nm. Both particle types accumulate in small clusters (Fig. 2B, D). ION@Dex falls in the size range found in the literature between 3 nm and 13 nm [19,20,66]. According to Kayal et al., the average diameter of ION@PVA lies in the range of 10–15 nm, which can be compared with the synthesized particles [28]. ION@PLGA has a d_TEM_ = 9.5 nm. The Scherrer diameter could not be calculated because the thick coating leads to small reflexes even with intense measurement time (Equation S1). The PLGA-coated particles show smaller clusters and even some single ION@PLGA (Fig. 2F).

In the human body, multiple media with different pH values can be found, ranging from the acidic gastric system with a pH of 2 over pH 4–5 for the lysosomal or endosomal fluid up to the neutral pH of 7.4 of the cellular fluid or blood [35,36,67]. pH value and ionic strength can highly influence the colloidal stability of nanoparticles and therefore affect their hydrodynamic diameters [51]. DLS and zeta potential were used to analyze the surface charge and colloidal stability. Between a zeta potential range of −10 mV and +10 mV, nanoparticles are considered unstable and tend to agglomerate strongly [11].

For ION@Dex, ION@PVA, and ION@PLGA, the hydrodynamic diameters are analyzed in dependence on the pH in water. ION@Dex agglomerated around the IEP of 6.94 with a hydrodynamic diameter of 1330 nm (Table 1, Fig. 2A). Unterweger et al. determined an IEP of 4.60 for dextran-coated IONs. But it should be noted that significantly higher amounts of dextran were used to synthesize the particles [68]. At zeta potentials above and below ±15 mV the ION@Dex had a Δd_DLS_ of 171 nm in water. Unterweger et al. also observed that ≥100 g L^−1^ dextran could prevent the particles from forming agglomerates [62]. Nevertheless, compared to BIONs with an IEP at 7.10, the dextran-coating led to a shift of the IEP to a lower pH value and better stabilization around the IEP [52,69]. ION@PLGA had an IEP at pH 2.88 (Fig. 2E, Table 1). The molecular weight of the lactic and glycolic chains, which influences the amount of carboxylic acid end groups, does affect the zeta potential [70]. Near the IEP, the particles formed agglomerates of 1061 nm. pH values > 5 led to higher zeta potentials > −22.6 mV. The stabilizing effect led to a hydrodynamic diameter of 242 nm at pH 7. Similar d_DLS_ between 100 and 250 nm have been observed in the literature [23,71,72]. Liang et al. used similar PLGA-coated nanoparticles with a hydrodynamic diameter of 220 nm to create a drug delivery system for paclitaxel. These particles are already in preclinical studies [72]. ION@PVA showed an IEP of 7.35, a value comparable to the literature (Fig. 2C, Table 1) [40]. The PVA coating led to the best stabilization, with an Δd_DLS_ = 198 nm, independent of the zeta potential and stable at the IEP. This effect can be attributed to the hydrophilic PVA chains [73,74].

**Table 1:** Mean diameter of ION@Dex, ION@PVA, and ION@PLGA determined via TEM and of XRD data with the Scherrer equation. For ION@PLGA the Scherrer diameter could not be calculated because the thick coating leads to small reflexes even with intense measurement time. IEPs of ION@Dex, ION@PVA, and ION@PLGA. Hydrodynamic diameters of BIONs, ION@Dex, ION@PVA, and ION@PLGA in SBF and human blood plasma at 37 °C.

Particles	TEM diameter d_TEM_ [nm]	Scherrer diameter d_Scherrer_ [nm]	Isoelectric point (IEP)	Hydrodynamic diameters in SBF [nm]	Hydrodynamic diameters in human blood plasma [nm]
BIONs				1905 ± 345	326 ± 17
ION@Dex	10.5 ± 2.1	8.6 ± 0.5	6.9	1416 ± 684	238 ± 18
ION@PVA	10.6 ± 2.1	8.8 ± 1.6	7.4	211 ± 7	238 ± 15
ION@PLGA	9.5 ± 2.1	–	2.9	841 ± 81	133 ± 1

**Fig. 3:**
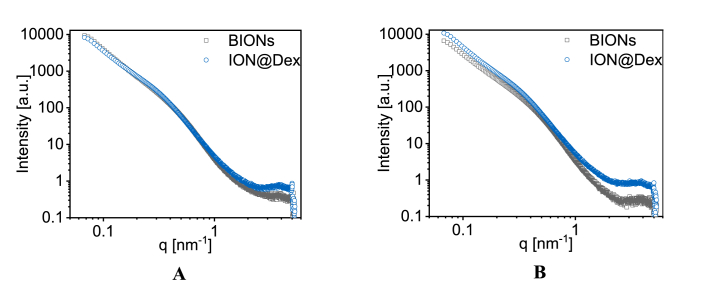
Small-angle X-ray scattering (SAXS) profiles of ION@Dex and BIONs at pH 4 (A) and 7 (B).

In addition to dynamic light scattering studies, we investigated small-angle X-ray scattering (SAXS) by taking BIONs and ION@Dex. By taking particles with and with coating we evaluate the agglomeration behavior and the primary particle size. SAXS profiles showed aggregation and primary particle sizes around 20 nm for all particles investigated (Fig. 3A and B). The highest aggregation is visible for BIONs at pH 7, which is in excellent agreement with DLS data and data from previous studies [52,75]. Even though this aggregation of nanomaterials is visible and makes it challenging to interpret the SAXS data, these results help to verify XRD data as well as TEM studies (Figs. 1C and 2). As complementary study ION@PVA are analyzed by a LDL assay.

Superparamagnetism is a crucial feature of IONs since it permits their application as a controllable drug delivery system, detection by MRI, or in hyperthermia therapy [11]. The saturation magnetization of the various coated particles was measured by superconducting quantum interference device (SQUID) and plotted against the applied magnetic field strength (Fig. 1A). All plotted curves showed the typical sigmoidal shape of superparamagnetic particles with no magnetization at a magnetic field strength of 0.0 Oe [11,76]. The used BIONs have a saturation magnetization of ±67.0 emu g^−1^ [52]. A slightly lower saturation magnetization of 62.0 emu g^−1^ can be seen for the dextran-coated particles. The Langevin Mod fit shows that the particles have an almost ideal profile. ION@PVA showed a saturation magnetization of ±56.7 emu g^−1^ and a curve that differs from the ideal fit above a magnetic field strength of ±20.0 kOe. Other studies with ION@PVA demonstrated superparamagnetic behavior and reported decreasing saturation magnetization with the increasing PVA coating [28]. For the ION@PLGA, only a saturation magnetization of ±1.60 emu g^−1^ was reached. This data corresponds to the IR measurements where the high characteristic PLGA peaks indicated a thick polymer coating. In the literature, similar behavior of ION@PLGA can be found between Lee et al. with a saturation magnetization <0.1 emu g^−1^ and Wang et al. with 4.00 emu g^−1^ [77,78]. The space- and time-resolved extinction profiles (STEP) technology was used to understand the particle's stability and magnetophoretic behavior at pH 7.4 (Fig. 1B). The sedimentation rates in a magnetic field increase with higher agglomeration and higher saturation magnetization of the particles [75]. In comparable conditions, BIONs sank with a sedimentation velocity of 1.2 mm s^−1^ [52]. As ION@Dex formed large agglomerates at physiological pH values, ION@Dex sank faster with a velocity of 2.2 mm s^−1^. However, BIONs as well as ION@Dex particles show a significantly lower sedimentation velocity without magnetophoretic sedimentation. (Fig. S3). ION@PLGA had sedimentation rates at 64.1 μm s^−1^, which fits the low saturation magnetization. PVA is known to stabilize the IONs highly, so even though it has a higher magnetization, its sedimentation velocity with 41.0 μm s^−1^ was comparably low as IONs@PLGA [73]. The specific surface area of the BIONs as well as of the dextrane coated particles is in the range of 100 m^2^ g^−1^ which corresponds to the particle size (Fig. S4).

FT-IR spectroscopy and XRD verified the successful synthesis. The coating material and thickness influenced the particles’ size, surface properties, saturation magnetization, and magnetophoretic behavior of the IONs. PVA coating showed the best stabilization of the IONs in a broad pH range and around the IEP.

The cytocompatibility of BIONs ION@Dex, ION@PVA, and ION@PLGA was determined by XTT assay after direct contact with HUASMCs after one and three days (Fig. 1D). The particles did not influence the cell morphology (Fig. S5). All particles show more than 70% viability compared to the negative control. ISO-10993 suggests this threshold for cytocompatibility. BIONs have been previously analyzed under the same conditions for their cell viability at a lower concentration of 0.08 g L^−1^ and show good cytocompatibility as well [52]. The cytocompatible behavior also fits other laboratory experiments for different coated ions with smooth muscle cells. E.g., Zhang et al. showed a minor decrease in cell viability for ION@DMSO, ION@APTS, or ION@Glu [79]. The cytocompatibility of BIONs and the different coated particles gives them the potential for application in nanomedicine.

**Fig. 4:**
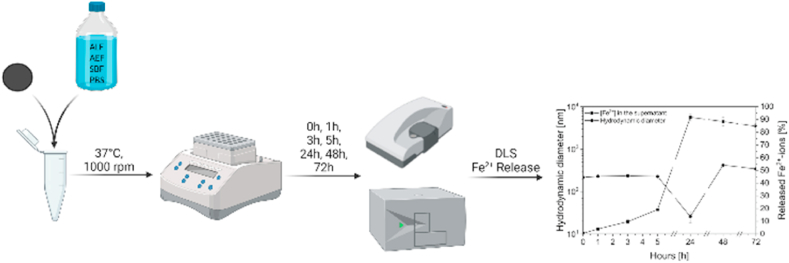
Schematic overview of the agglomeration and Fe^2+^-release study over 72 h with BIONs, ION@Dex, ION@PVA, and ION@PLGA.

To better understand the impact of coating materials on the IONs degradation and agglomeration profile, BIONs, ION@Dex, ION@PVA, and ION@PLGA were incubated in SBF, ALF, AEF, and PBS for 72 h at 37 °C (Fig. 4).

In contrast to Rabel et al., a faster protocol was used here, which shortens the protocol to three days (vs. 28 days) [36]. Faster shaking speeds (1000 rpm vs 110 rpm) ensure that the particles are kept in suspension and therefore have more contact with the medium.

**Fig. 5:**
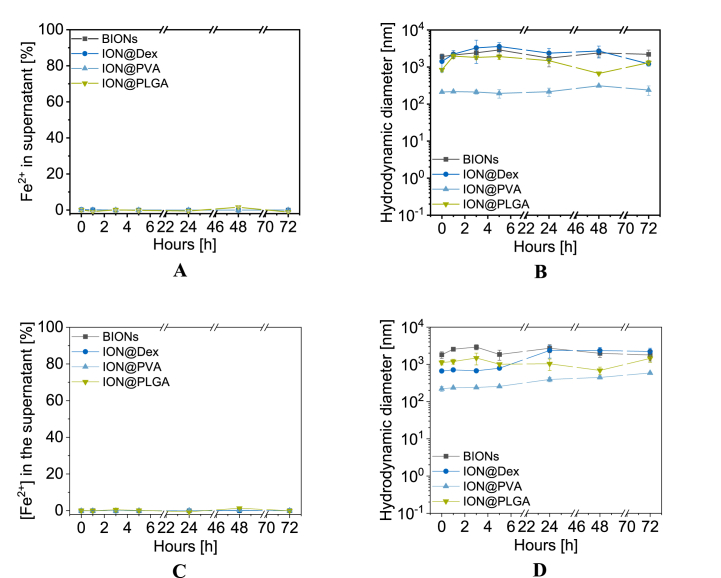
Fe^2+^ release profiles in SBF (A) and PBS (C) and agglomeration in SBF (B) and PBS (D) for BIONs, ION@Dex, ION@PVA, and ION@PLGA for 72 h at 37 °C (1000 rpm).

The initial contact with the body after injection in the bloodstream was analyzed in SBF (Fig. 5A snd B, Table S1). Over this period, regardless of particle composition, no iron release was detected in the supernatants of the particle solutions. This behavior was also observed in the literature and can be explained by the low solubility of the particles in physiological conditions [36]. Rabel et al. did not observe any dissolution of organic (Starch, Dextran, Chitosan) and inorganic (Silica) coated particles over 28 days [36]. Compared to the hydrodynamic diameter of BIONs (504 ± 10.5 nm) in d_H2O_ (pH 7.4), in SBF, a 3.77 times higher agglomeration with an initial hydrodynamic diameter of 1905 ± 345.2 nm occurred. Over 72 h, hydrodynamic diameters of BIONs remained at 1.00 μm–2.00 μm, indicating constant agglomeration of the BIONs. At physiological pH, the BIONs with an IEP at 7.10 don't show a strong surface charge and form agglomerates accordingly [52]. Furthermore, high electrolyte concentrations in the medium, such as sodium, calcium, chloride, and hydrogen phosphate, increase the aggregation [36,80]. ION@Dex already showed large hydrodynamic diameters in water at pH 7.17 of 1330 nm and colloidal instability around its IEP of 6.94. In SBF, the particles demonstrated almost similar initial agglomeration with hydrodynamic diameters of 1416 nm compared to diameters in water. After 72 h, hydrodynamic diameters increased up to >2.00 μm. A thicker dextran coating could decrease the agglomeration [81]. ION@PLGA showed an agglomeration over time from 841 nm to 1311 nm after three days. Even though the particles had a stable colloidal behavior in water (242 nm) at comparable pH values because of its IEP at acidic pH values, the salts induce 5.4 times higher agglomeration. The hydrodynamic diameters of ION@PVA are minimally influenced by SBF, leading to agglomerates of 240 nm after 72 h. This agglomeration is comparable to water at 205 nm. The long polymer chains support the colloidal stability [36,73]. PVA is known to form hydrogen bonding between the polymer chains resulting in a hydrogel structure, embedding the particle, and responsible for steric stabilization [81]. SBF simulates the salt composition, concentration, and pH value, whereas proteins and viscosity of the blood were not considered. Human blood plasma contains proteins (Albumin, IgG, Transferrin), glucose, mineral ions, hormones, carbon dioxide, and blood cells [82]. To better understand the effect of those additional components, the hydrodynamic diameters in SBF and in human plasma were compared (Table 1, Fig. S6).

The agglomerates of BIONs, ION@Dex, and ION@PLGA highly decreased in human blood plasma. The hydrodynamic diameters of ION@PVA remained constant. All particles had diameters <350 nm, and all coatings led to a colloidal stabilization. This effect can be caused by the higher viscosity of around 1.44 mPa and the binding of plasma proteins [40,75,83]. Because no degradation takes place, the small hydrodynamic diameters of the coated IONs should ensure prolonged blood circulation times.

PBS buffer (pH 7.40) was used to simulate the cytoplasm of the cell (Fig. 5C and D, Table S2) [40]. The Fe^2+^-release experiments showed identical results as in SBF. The IONs don't dissolve at physiological pH values. Congruent to SBF, BIONs agglomerate in larger hydrodynamic diameters, with a Δd of 2227 nm, then the coated particles. Hydrodynamic diameters of ION@Dex remained smaller (788 nm) for 5 h, whereas almost identical hydrodynamic diameters of ≥2.00 μm to BIONs occurred after 24 h. The effect can be attributed to the change in buffer composition since the pH value was not modified. Compared to SBF, an amount of potassium phosphate (1.20 g L^−1^) and sodium phosphate (7.20 g L^−1^) can be found in PBS. Therefore, it can be assumed that phosphate ions bind on the surface of ION@Dex and stabilize the particle in the first 5 h. Almasri et al. can verify the stabilizing effect of absorbed phosphate ions [84]. ION@PVA starts with similar agglomerate sizes of 220 nm compared to SBF. After 5 h, the particles formed larger hydrodynamic diameters up to 588 nm. ION@PLGA was less influenced over time and showed sizes between 1120 and 1440 nm.

Both buffers at physiological pH values did not lead to ION degradation. The salt concentration, viscosity, and protein content influenced the agglomerate size; all coatings did decrease this size. At the same time, only the PVA coating showed distinct smaller hydrodynamic diameters than the other coatings. All particles experienced distinct smaller hydrodynamic diameters in human blood serum.

In the cell, the first stage of degradation of foreign material is found in endosomes and was simulated by AEF (Fig. 5) [41]. The pH of the endosome is decreased to 5.5 by proton pumps that influx the H^+^ [85]. Here the first stage of degradation of foreign material from the cell occurs. Accordingly, the particles' initial degradation by released iron ions can be observed. BIONs experienced a constant iron ion release of up to 15.3% after 72 h. The thick PLGA coating can degrade to lactic acid and glycolic acid in acidic media [[86], [87], [88]]. Afterward, the IONs are dissolved. After 72 h, 9.56% of iron ions are released from ION@PLGA. ION@Dex and ION@PVA dissolve faster than BIONs. A dissolution of 20.4% and 21.9% is reached. Similar trends have been observed in the literature for DEAE-Dextran, and chitosan-coated IONs [36]. These particles protonated at acidic pH values and attracted more water and dissolution agents, leading to faster iron ion release [36]. Rabel et al. showed dissolution of ∼20% after 14 days, indicating that the accelerated mixing (1000 rpm vs. 110 rpm [36]) speeds up the degradation 4.6 times in AEF. This effect can be attributed to the shear forces exerted by the shaking and the better mixing of the particles. At low mixing rates, the particles sediment to the bottom. As a result, the individual particles are more difficult to access and dissolve slowly because a larger agglomerate has a smaller accessible surface area. Lanzl et al. found that physicochemical properties such as morphology, size, and agglomeration behavior also influence the dissolution profile of the particles [89]. Thus, 40 nm-sized particles dissolve up to ten times slower than smaller particles in pH values between 1.00 and 7.00 [89]. In the first 24 h, the dissolution of BIONs can be followed by DLS. Hydrodynamic diameters decrease from 82.2 nm to 65.1 nm (6.80% Fe^2+^-release). Afterward, the particle agglomerated to a size of 346 nm after 72 h. The large agglomerates were dissolved slower. Gutierrez et al. have shown that the degradation of BIONs increases with decreasing pH [35]. Furthermore, an acidic pH reduces the protection of organic shells against chelating components of AEF such as citrate and lactate [35,36]. The dissolution of iron oxides in organic acids such as citrate is a multi-step process. By chemisorption, the acid adsorbs onto the iron oxide surface. Here, the particle surface's Lewis base/acid properties are involved. Then, non-reductive dissolution can occur. Thus, iron-ligand complexes dissolve from the surface as a whole. This process is characterized by high activation energy, achieved only at high temperatures [90]. In these experiments, at 37 °C, reductive dissolution is more dominant. Fe^2+^ ions of magnetite dissolve from the crystal and accumulate in the solution [[90], [91], [92]]. The carboxylic acids of citrate can complex with iron ions, similar to EDTA. The chelate complexes promote the dissolution of iron oxides [90]. ION@PLGA demonstrated initial agglomeration with hydrodynamic diameters of 824 nm, which is 3.4 times higher than in water. After 24 h, the size decreased to 384 nm. Similar to BIONS, the PLGA-coated particles started to agglomerate in the last two days (1108 nm). Without additional agglomeration, ION@Dex decreased its hydrodynamic diameters from 127 nm to 25.8 nm after 72 h.

The initial size is comparable to the agglomeration in water at acidic pH values. The results of ION@PVA revealed constant hydrodynamic diameters of 200 nm–300 nm. This behavior does not correspond to the degradation study. It can be assumed that larger agglomerated particles degraded slower and were detected in DLS.

**Fig. 6:**
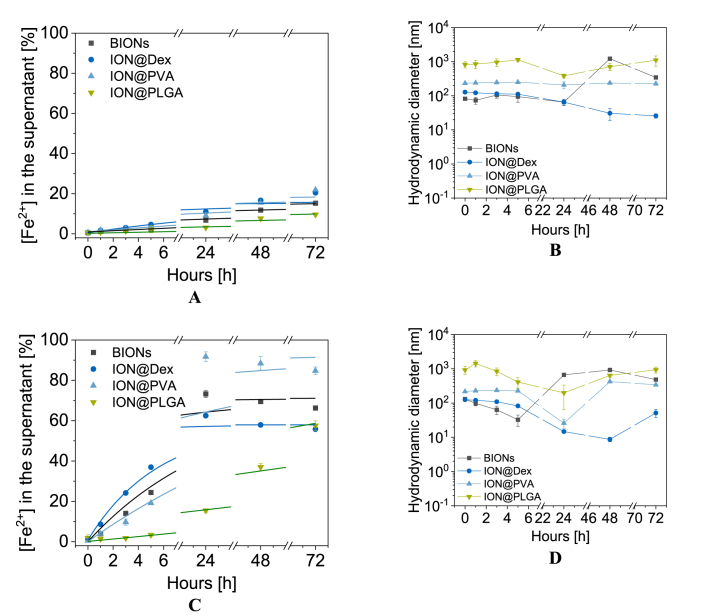
Fe^2+^ release profiles in AEF (A) and ALF (C) described with a first order kinetic and agglomeration in AEF (B) and ALF (D) for BIONs, ION@Dex, ION@PVA, and ION@PLGA for 72 h at 37 °C (1000 rpm).

ALF simulated the degradation of the particles in the lysosome with an acidic pH value of 4.5 (Fig. 6A and B). The initial size is comparable to agglomeration in water at acidic pH values. The results of ION@PVA revealed constant hydrodynamic diameters of 200 nm–300 nm. This behavior does not correspond to the degradation study. It can be assumed that larger agglomerated particles degraded slower and were detected in DLS (Fig. 6C and D, Table S5) [41]. BIONs experience a fast dissolution in the first 24 h, up to 73.3% iron ion release migrating into a plateau. The dissolution of ION@PVA starts slower in the first 5 h but then speeds up and reaches 91.0%. A plateau setting suggests that these particles are almost entirely degraded [36]. Due to the IEP of ION@PVA (7.35), the surface is positively charged and thus attracts more water and solubilizing agents [36]. In the beginning, ION@Dex experienced a faster dissolution than BIONs, slowing down after 5 h to a maximal degradation of 62.5% (24 h). ION@PLGA is dissolved up to 57.5% after 72 h. As the coating dissolved first, the curve experienced a smaller slope. In ALF, the particles dissolved four times faster than in AEF, which was also reported by Guiterrez et al. and Rabel et al. for differently coated IONs [35,36].

Hydrodynamic diameters of BIONs decreased rapidly from 130 nm to 32.6 nm after 5 h. Afterward, the diameters increased and stayed constant for the next days at a Δd ∼692 nm. The data verify that the decrease in hydrodynamic diameters can visualize the degradation in the acidic surrounding. The remaining 26.7% of particles seem to form huge agglomerates. As larger particles take longer to dissolute, the Fe^2+^ release slowed down and reached a plateau. Comparable to AEF, ION@PLGA initially formed huge agglomerates of 922 nm. The hydrodynamic diameters constantly decreased until 199 nm at 24 h, congruent to the degradation profile. Afterward, the sizes increased again up to 932 nm. Compared to AEF (384 nm, 24 h), the hydrodynamic diameters of ION@PLGA showed a higher and faster decrease in ALF (198 nm, 24 h). ION@Dex showed an initial size of 125 nm. ION@Dex's hydrodynamic diameters decreased from 125 nm to 8.71 nm in 48 h. In the last hour, the remaining particles formed aggregates of 51.5 nm. Similar behavior was observed for the PVA-coated particles. The size decreased from 217 nm to 25.8 nm in the first 24 h, while afterward, the IONs showed agglomerates of 342 nm.

In summary, the organic coatings did stabilize the particles. The acidic environment did induce degradation. In ALF, the particles dissolved faster than in AEF. All particles except ION@PLGA reached a plateau in 72 h due to the formation of big agglomerates. ION@PVA experienced the fastest iron ion release. ION@PLGA had the highest initial agglomeration in the acidic media.

**Fig. 7:**
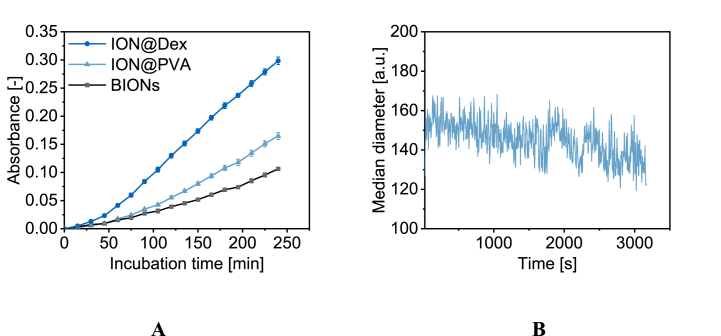
Diene oxidation of dissolved nanoparticles (48 h at pH 4.5 in acetate buffer) in acetate buffer (pH 4.5) (A). Standard deviation derives from at least four independent experiments. Dissolution of ION@PVA particles in acetate buffer at pH 4.5 (B).

Oxidative stress induced by the dissolved particles after 48 h was emulated with an low-density lipoprotein (LDL) assay. Due to the thick coating of ION@PLGA that leads to different mass balance only BIONs and the thin coatings (PVA, Dex) were used. Here oxidation is fastest for dissolved ION@Dex particles and slowest for dissolved BION particles in an acetate buffer. Due to oxidative stress by reactive oxygen species (ROS) of the dienes, the oxidation was monitored over 240 min (Fig. 7A). The results indicate that the coating plays a role in the dissolution of the magnetic particles and in the oxidation state and therefore influences the oxidation behavior. Here, the dextran-coated particles, which also tend to be dissolved fastest in AEF, show the highest oxidation kinetic. Interestingly, the naked BIONs demonstrate the slowest kinetic, which is in good agreement with the dissolution of these particles.

The dissolution kinetic of ION@PVA shows slight aggregate size decreases with the optofluidic force induction measurements (Fig. 7B). These measurements indicate a direct size decrease even though the exact hydrodynamic diameter cannot be reflected (Fig. S5).

## 4 Conclusion

Three different commonly used organic coatings on IONs, PLGA, PVA, and Dex were successfully synthesized. The coatings influence the particles' properties. While all had similar d_TEM_ the IEP point influenced the colloidal stability. ION@PVA showed the least agglomeration over a pH range from 4 to 10. SAXS profiles, as an alternative method, could visualize aggregation and primary particle sizes around 20 nm for BIONs and ION@Dex. The coating thickness affected the saturation magnetization, whereas the agglomeration also influenced the sedimentation velocity in a magnetic field. All particles, including BIONs, showed good cytocompatibility (>70%) over three days in smooth muscle cells. The experimental setup for long-term agglomeration and degradation studies did speed up the process by a factor of 4.6, allowing fast screening of multiple candidates and thus can shorten the preclinical phase. Furthermore, it was ensured the particles didn't sediment during the experiment. The investigated particles all have different colloidal stability and dissolution profiles. In SBF and PBS, none of the investigated particles dissolved. ION@PVA showed the least agglomeration. A first degree of degradation of the particles is visible in AEF, which confirms the pH dependence of the dissolution. The IONs did dissolve faster in ALF, with the degradation rate decreasing with the shrinking size, leading to a plateau. The fastest Fe^2+^ release could be measured for ION@PVA in ALF, while ION@PLGA experienced the lowest degradation. The oxidation kinetic of BIONs was slower than ION@Dex and ION@PVA, fitting to the degradation results in AEF. This study provides essential insights into the agglomeration and degradation profile and the oxidative stress of IONs with standard coatings for medical applications. The used analytical setup combining DLS, phenanthroline assay, SAXS, and LDL assay is ideal for a fast preclinical study of new IONs, giving often neglected yet crucial information about the behavior and toxicity of nanoparticles in the human body. With this study we want to emphasize the dissolution of nanomaterials and the potential use of generally cytocompatible iron oxide particles for ferroptosis applications.

## Author contribution statement

Chiara Turrina: Conceived and designed the experiments; Performed the experiments; Analyzed and interpreted the data; Wrote the paper.

Anna Klassen, Davide Milani, Diana Rojas-Gonzalez, Gerhard Ledinski, Doris Auer, Barbara Sartori: Performed the experiments; Analyzed and interpreted the data.

Gerhard Cvirn: Analyzed and interpreted the data; Wrote the paper.

Petra Mela, Sonja Berensmeier: Analyzed and interpreted the data; Contributed reagents, materials, analysis tools or data; Wrote the paper.

Sebastian Schwaminger: Conceived and designed the experiments; Performed the experiments; Analyzed and interpreted the data; Contributed reagents, materials, analysis tools or data; Wrote the paper.

## Funding

We appreciate the support from TUM International Graduate School of Science and Engineering (IGSSE). The funders had no role in the design of the study, the collection, analysis, and interpretation of data, the writing of the manuscript, or the decision to publish the results.

## Data availability statement

Data will be made available on request.

## Declaration of competing interest

The authors declare that they have no known competing financial interests or personal relationships that could have appeared to influence the work reported in this paper.
