# Supplementary material for: Superparamagnetic iron oxide nanoparticles for their application in the human body: Influence of the surface
Source: Heliyon. 2023 May 25;9(6):e16487. doi: 10.1016/j.heliyon.2023.e16487 (PMC10238907; doi:10.1016/j.heliyon.2023.e16487)
Supplement: Multimedia component 1 [file mmc1.docx]

**Supplementary Material:**

**Screening of superparamagnetic iron oxide nanoparticles for their application in the human body: Influence of various coatings**

Chiara Turrina,^a^ Anna Klassen,^a^ Davide Milani,^a^ Diana M. Rojas-González,^b^ Gerhard Ledinski,^c^ Doris Auer,^d^ Barbara Sartori,^e^ Gerhard Cvirn,^c^ Petra Mela,^b^ Sonja Berensmeier,^a^ and Sebastian P. Schwaminger^a,c,f,*^

^a^Chair of Bioseparation Engineering, Department for Engineering and Design, Technical University of Munich, Germany

^b^Chair of Medical Materials and Implants, Department for Engineering and Design, Technical University of Munich, Germany

^c^Division of Medicinal Chemistry, Otto Loewi Research Center, Medical University of Graz, Austria

^d^Division of Medical Physics and Biophysics, Gottfried Schatz Research Center, Medical University of Graz, Austira

^e^Institute of Inorganic Chemistry, Graz University of Technology, Stremayrgasse 9/IV, Graz, 8010, Austria

^f^BioTechMed-Graz, Austria

**Figure S1**:IR spectra of ION@PLGA, ION@PVA, and ION@Dex.

|   **A** |   **B** |
| --- | --- |

**Figure S2**:X-ray diffractogram of A) ION@PVA and B) ION@PLGA.

$$L=\frac{K*\lambda}{\beta*cos(\theta)}$$

Equation S1: Scherrer equation was used to calculate the particle size L of the magnetite crystal. The Scherrer shape factor K has a constant value of 0.89. The X-ray wavelength l is 0.07093 nm. The Bragg angle θ_0_ and the full half-width of the reflection Δ2θ are calculated using Origin software. The two largest reflections of the plane (311) and (440) were used

**Table S1**: Composition of 1 L 50 mM PBS buffer (pH 7.4).

| **Component** | **Mass** |
| --- | --- |
| NaCl | 40.0 g |
| KCl | 1.00 g |
| NaH2PO4 | 7.20 g |
| KH2PO4 | 1.20 g |

**Table S2**: Composition of ALF and AEF per liter according to Marques et al. [1] pH adjusted to 4.5 for 4.5 for ALF and 5.5 for AEF.

| **Component** | **Mass** |
| --- | --- |
| Calcium chloride dihydrate | 0.13 g |
| Citric acid | 20.8 g |
| Glycerol | 0.06 g |
| Magnesium chloride | 0.05 g |
| Sodium citrate dihydrate | 0.08 g |
| Sodium chloride | 3.21 g |
| Sodium phosphate heptahydrate | 0.18 g |
| Sodium lactate | 0.09 g |
| Sodium hydroxide | 6.00 g |
| Sodium pyruvate | 0.09 g |
| Sodium sulfate | 0.04 g |
| Sodium tartrate dihydrate | 0.09 g |
| Formaldehyde 37% | 2.70 mL |

**Table S3**: Composition of SBF per liter according to Marques et al. [1] pH adjusted to 7.4.

| **Component** | **Mass** |
| --- | --- |
| Calcium chloride dihydrate | 0.38 g |
| Magnesium chloride | 0.15 g |
| Potassium chloride | 0.23 g |
| Potassium phosphate | 0.14 g |
| Sodium chloride | 8.04 g |
| Sodium bicarbonate | 0.36 g |
| Sodium sulfate | 0.07 g |
| Tris(hydroxymethyl)aminomethane | 6.12 g |
| 1M hydrochloric acid | 39.0 mL |

**Table S4**: Phenanthrroline assay: Fe^2+^-stock solution.

| **Component** | **Mass** |
| --- | --- |
| FeCl_2_(H2O)_4_ | 35.5 mg |
| dH_2_O | 100 mL |

**Table S5**: Phenanthroline assay: 10% ascorbic acid solution.

| **Component** | **Mass** |
| --- | --- |
| L-ascorbic acid | 5.01 mg |
| dH_2_O | 50.0 mL |

**Table S6**: Phenanthroline assay: Acetic acid buffer (pH 4.5).

| **Component** | **Mass** |
| --- | --- |
| Acetic acid | 15.0 mL |
| 1M NaOH | 130 mL |
| dH_2_O | 115 mL |

**Table S7**: Phenanthroline assay: Phenanthroline solution.

| **Component** | **Mass** |
| --- | --- |
| Phenanthroline | 2.50 mL |
| dH_2_O | 50.0 mL |

**Figure S3**: Cumulative velocity distribution at pH 7 in water, at room temperature with (M) and without magnetopheretic sedimentation.

**Figure S4**: Nitrogen adsorption isotherm on BION and ION@Dex particles at 77 K.

**Figure S5**: Hydrodynamic diameters of BIONs, ION@PLGa, IONqPVA, and ION@Dex in SBF and human blood plasma (HP).


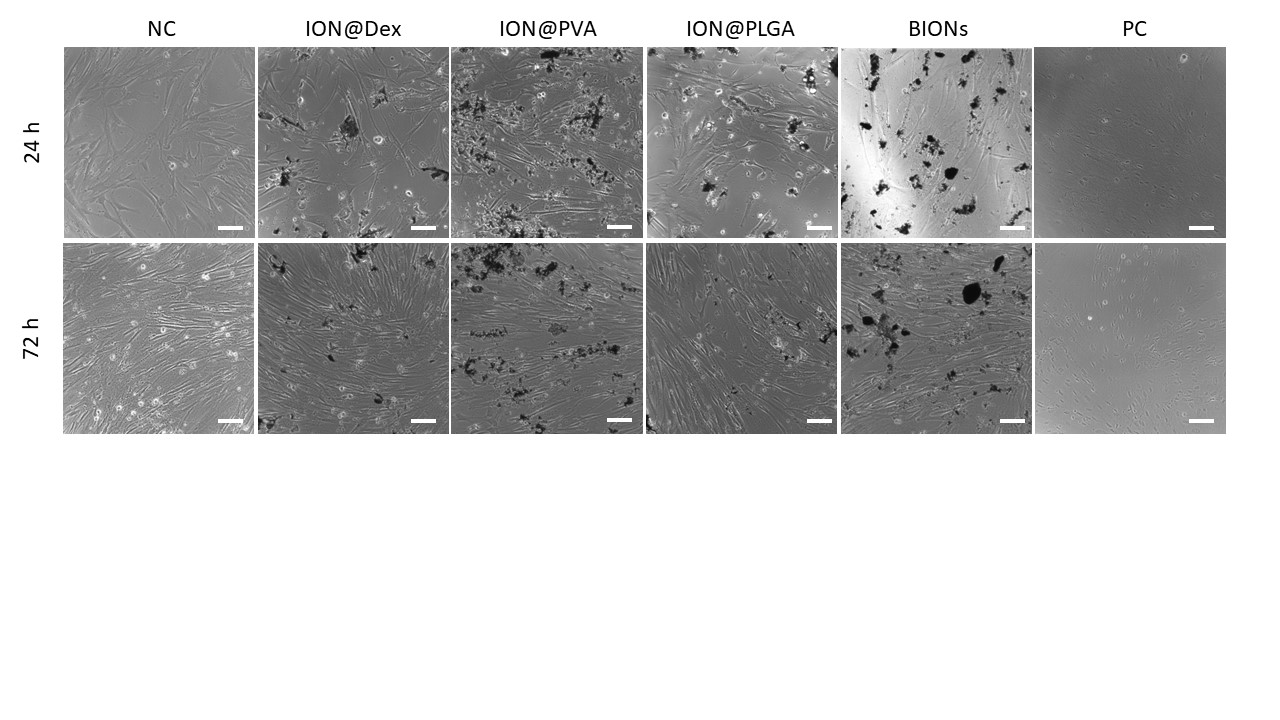


**Figure S6**: Phase contrast images of the cells incubated with IONs@CMD and BIONs after 24 and 72 hours for the cytocompatibility assay. Scale bar: 100 µm.

References

[1] Margareth R. C. Marques, R. Loebenberg, M. Almukainzi, Simulated Biological Fluids with Possible Application in Dissolution Testing, Dissolution Technologies (2011) 15–28. https://doi.org/10.14227/DT180311P15.
